# Supplementary material for: REC drives recombination to repair double-strand breaks in animal mtDNA
Source: J Cell Biol. 2022 Nov 10;222(1):e202201137. doi: 10.1083/jcb.202201137 (PMC9652705; doi:10.1083/jcb.202201137)
Supplement: Table S1 — a list of candidate genes examined for the mitochondrial enrichment of their protein products when overexpressed in Drosophila S2R+ cells (Fig. 1 A). [file JCB_202201137_TableS1.docx]

**Supplementary Tables S1**

**A list of candidate genes examined for the mitochondrial enrichment of their protein products when overexpressed in *Drosophila* S2R+ cells (Fig. 1A).**

| **Gene** | **Description** | **Human ortholog** | **MitoProt score** | **S2R+ subcellular enrichment** |
| --- | --- | --- | --- | --- |
| *rec* | Meiotic MCM protein | MCM8 | 0.98 | Nuclear and mitochondrial |
| *Hdm* | Meiotic Single Strand Binding protein | MEIOB | 0.99 | Mitochondrial |
| *CG12728* | NEHJ Protein | XLF | 0.18 | Nuclear and mitochondrial |
| *XRCC4* | NEHJ Protein | XRCC4 | 0.835 | Cytoplasmic |
| *DNA2* | Helicase/ Endonuclease | DNA2 | 0.23(B) / 0.31(C) | Nuclear |
| *Pif1* | Helicase | PIF1 | 0.04 | Nuclear |
| *mre11* | HR and DSB Repair Nuclease | MRE11 | 0.06 | Nuclear and cytoplasmic |
| *Rrp1* | Endonuclease/ Endonuclease | APE1 | 0.03(A) / 0.03(B) | Nuclear |
| *Spn-A* | HR and DSB Repair Nuclease | RAD51 | 0.03(A) / 0.43(B) | Cytoplasmic |
| *spn-D* | HR and DSB Repair Nuclease | RAD51C | 0.43 | Cytoplasmic |
| *spn-B* | Meiotic and Mitotic Endonuclease | XRCC3 | 0.04 | Cytoplasmic |
| *parp* | DNA PARylation Enzyme | PARP1 | 0.01 | Nuclear |
| *mms19* | NER and FeS Cluster Assembly Protein | MMS19 | 0.03 | Cytoplasmic |
| *CG9601* | DNA Kinase | PNKP | 0.5 | Nuclear and cytoplasmic |
| *Fen1* | Flap Endonuclease | FEN1 | 0.28 | Nuclear |
| *Ercc1* | Subunit of Meiotic Nuclease | ERCC1 | 0.12 | Nuclear |
| *XLF2* | NEHJ Protein | XLF | 0.28 | Nuclear |
| *Okra* | Meiotic Helicase | RAD54L | 0.87 | Nuclear |
| *Rad51D* | HR and DSB Repair Nuclease | RAD51D | 0.33 | All over |
| *Gen* | Endonuclease | GEN1 | 0.013 | Cytoplasmic |
| *mus81* | Crossover Endonuclease | MUS81 | 0.06 | Cytoplasmic |
| *mei-217* | Involved in Female Meiosis with mei218 | - | 0.03 | Nuclear |
| *mei-218* | Involved in Female Meiosis with mei217 | MCMDC2 | 0.11 | Nuclear punctae |
| *Rad23* | Proteosome Binding and NER Protein | RAD23A/B | 0.06(A) / 0.12(B) | Nuclear |
| *CG10694* | Proteosome Binding and NER Protein | RAD23A/B | 0.37 | All over |
| *mus312* | Scaffolding for Endonucleases | SLX4 | 0.73(A) / 0.55(D) / 0.77(E) | Nuclear |
| *Nse1* | RING Finger Protein | NSMCE1 | 0.325 | All over |
| *WRNexo* | Exonuclease | WRN | 0.17 (A)/ 0.13(B) / 0.26(C) | Nuclear |
| *mei-9* | Subunit of Meiotic Nuclease | ERCC4 | 0.02 | Cytoplasmic punctae |
| *CG14443* | RNA Helicase | DDX5 | 0.01 | Nuclear |
| *RecQ5* | Helicase | RECQL5 | 0.07(A) / 0.09(B) / 0.01(C) | All over |
| *CG6171* | DNA Repair Endonuclease/ Exonuclease | APLF | 0.35(A) / 0.35(B) | Nuclear |
| *FancI* | Fanconi Anemia Protein | FANCI | 0.18 | Nuclear |
| *FancL* | Fanconi Anemia Protein | FANCL | 0.09(A) / 0.09(B) | Nuclear and cytoplasmic |
| *Xpac* | NER Protein | XPA | 0.04 | Nuclear |
| *Sws1* | HR Regulator | RAD1 | 0.13 | Nuclear and cytoplasmic |
| *tdrd3* | RNA Binding and Scaffolding Protein | TDRD3 | 0.052 | Cytoplasmic |
| *Pcm* | Exoribonuclease | XRN1 | 0.68(A) / 0.68(B) | Cytoplasmic |
| *CG30094* | Homolog of Yeast Sea3 | - | 0 | Nuclear and cytoplasmic |

**A list of primers used for the generation of *Drosophila* stocks**

| **Primer name** | **Sequence** |
| --- | --- |
| F1 REC-Halo | GAGCTCGGTACCCGGGGATCGTGTTGGGCGTGATTGTACGACC |
| R1 REC-Halo | CCTGAACCACCGCTACCACCGTCAGGGACCACTTCGTACATGTTTGC |
| F2 REC-Halo | TGTACGAAGTGGTTCCTGACGGTGGTAGCGGTGGTTCAGGTG |
| R2 REC-Halo | CTATATCGTTTGTCATTTGCCTAGGAAATCTCCAGAGTAGACAGCC |
| F3 REC-Halo | CTATATCGTTTGTCATTTGCCTAGGAAATCTCCAGAGTAGACAGCC |
| R3 REC-Halo | CAGGTCGACTCTAGAGGATCGCTTAAGCCTCCCAGCTTCATACC |
| F2 REC-Flag | TGTACGAAGTGGTTCCTGACGGTGGTAGCGACTACAAAGACCATG |
| R2 REC-Flag | CTATATCGTTTGTCATTTGCCTAGGACCGGTGCTTGTCATCG |
| F1 rec delMTS | GAGCTCGGTACCCGGGGATCGGATGATGATTCGGATGACGATGG |
| R1 rec delMTS | CATTTCAGCTGGGGAAAGTTTACAA |
| F2 rec delMTS | AACTTTCCCCAGCTGAAATGCCCAACCAAGGGGAACCTGGAG |
| R2 rec delMTS | CAGGTCGACTCTAGAGGATCCGTGGGCACCACGTTCAGCG |
| F1 HDM-Halo | GAGCTCGGTACCCGGGGATCATGGCAAGGCGTATTAAGTTCCAGC |
| R1 HDM-Halo | CCCTGGAAATACAGGTTTTCACTAGTGTTGAAAACGACCATATTTGCGACAAGC |
| F2 HDM-Halo | GAAAACCTGTATTTCCAGGGAGCAGCCTCGGGTAGCGGTGGTTCAGGTGGTAG |
| R2 HDM-Halo | TGGGACCACCCCACATACACCTAGGAAATCTCCAGAGTAGACAGC |
| F3 HDM-Halo | GTGTATGTGGGGTGGTCCCATC |
| R3 HDM-Halo | CAGGTCGACTCTAGAGGATCGCGTCAGCAACTCCAATGGAACC |
| F1 PolG1-Flag | AAACGACGGCCAGTGAATTCGAGCTCGGTACCCGGGGATC |
| R1 PolG1-Flag | CTTTGTAGTCGCTACCACCACTCTTCTTTATCCAATCCCACTG |
| F2 PolG1-Flag | GTGGTGGTAGCGACTACAAAGACCATGACGGTG |
| R2 PolG1-Flag | GTATTTGGCAGCTAGGACCGGTGCTTGTCATCGTC |
| F3 PolG1-Flag | CGGTCCTAGCTGCCAAATACTAGAGATTAAATCATAAGTAC |
| R3 PolG1-Flag | ACACTTGGCGTAATCATGGTCATAGCTGTTTCCTG |
| F1 XLF1-Halo | GAGCTCGGTACCCGGGGATCGGTGAAATAGTGTGAGTGCAACACG |
| R1 XLF1-Halo | TTTTTGCGCTAACTTATTGAT |
| F2 XLF1-Halo | TCAATAAGTTAGCGCAAAAAGGTGGTAGCGGTGGTTCAGGTG |
| R2 XLF1-Halo | CTGAGATCGTTTCCAAATTAGGAAATCTCCAGAGTAGACAGCC |
| F3 XLF1-Halo | TTTGGAAACGATCTCATTCTAAATGG |
| R3 XLF1-Halo | CAGGTCGACTCTAGAGGATCCGATGGTGATGTATAAAGATTCC |
| F4 XLF1-Halo | AACCAATTCAGTCGACTGGTGAAATAGTGTGAGTGCAACACG |
| R4 XLF1-Halo | ATAAATCAGCACAGCTTTGATATCTTCCAGCACAG |
| F5 XLF1-Halo | CTGTGCTGATTTATTCAACTGAACGCATCG |
| R5 XLF1-Halo | ATCTCGAGTGCGGCCGCGATGGTGATGTATAAAGATTCC |
| F1a SPN-B-Halo | GAGCTCGGTACCCGGGGATCCCTTATTGGATGCCCACTGTAGTTGCC |
| R1a SPN-B-Halo | CCTGAACCACCGCTACCACCCAGTCGACGCATTTTGGCGGGTGGGC |
| F2 SPN-B-Halo | GGTGGTAGCGGTGGTTCAGGTGGTAG |
| R2 SPN-B-Halo | CTAGGAAATCTCCAGAGTAGACAGCCAG |
| F3a SPN-B-Halo | CTACTCTGGAGATTTCCTAGTTGATTGAATTTATTCTGTTTATTTTGTTATCG |
| R3a SPN-B-Halo | CAGGTCGACTCTAGAGGATCCCAAAGTCTCCAAATCGCGGCAGTAAC |
| F1b SPN-B-Halo | AACCAATTCAGTCGACTCCTTATTGGATGCCCACTGTAGTTGCC |
| R1b SPN-B-Halo | GAAACTCAGCGAAATCATTAGGAGTCTCTGGTGAGTAAAGTATTTCC |
| F2b SPN-B-Halo | TAATGATTTCGCTGAGTTTCTTATCACCGCAGAAGGTGTGG |
| R2b SPN-B-Halo | TGCCGTTCCACATTTTAAATTCAACAAATTAAACG |
| F3b SPN-B-Halo | ATTTAAAATGTGGAACGGCAGAGCGTTCAATTAGCCATACG |
| R3b SPN-B-Halo | ATCTCGAGTGCGGCCGCCAAAGTCTCCAAATCGCGGCAGTAAC |
| F1 SPN-D-Halo | GAGCTCGGTACCCGGGGATCCCAATAAAATCGCGTCGCAGG |
| R1 SPN-D-Halo | CCTGAACCACCGCTACCACCTTCGGATTCTTTGATTAATCTACTCGTTCGCC |
| F3 SPN-D-Halo | TGTCTACTCTGGAGATTTCCTGATTCTACATCACTTTTACTTTGTG |
| R3 SPN-D-Halo | CAGGTCGACTCTAGAGGATCGATAGCTATAGCTTTCCGTCGGC |
| F1 MRE11-GFP | AAAGGAACCAATTCAGTCGACTTTGTACGGACTTAGCCACATC |
| R1 MRE11-GFP | CCACTACCACCTGAACCACCGCTACCACCATCGGAATCATCGGATATGACATAGCTGG |
| F2 MRE11-GFP | GGTGGTTCAGGTGGTAGTGGCGGCAGCGCCACCATGGTGAGCAAGG |
| R2 MRE11-GFP | CCGTGGACCGGTGCTTGTAC |
| F3 MRE11-GFP | TGTACAAGCACCGGTCCACGGGAAGTGGATCTGCTGGTAGTGC |
| R3 MRE11-GFP | TTAGGCATAATCCGGAACGTCGTAC |
| F4 MRE11-GFP | ACGTTCCGGATTATGCCTAATTTGAATTGCGGAGACGTTGTTTACCAC |
| R4 MRE11-GFP | TAGATATCTCGAGTGCGGCCCCAGTTCTTTCCGTCTGAGTGC |
| F1 NBS-GFP | AAAGGAACCAATTCAGTCGACGCCACTCGGCGCAATCCAATGCCACG |
| R1 NBS-GFP | CCTGAACCACCGCTACCACCAACACAGCTTACCATACCATCAGGCAGCGGAAGACTCTT |
| F2 NBS-GFP | GGTGGTAGCGGTGGTTCAGGTGGTAGTGGCGGCAGCATGGTGAGCAAGGGCGAGGAGCTG |
| R2 NBS-GFP | TCACGTGGACCGGTGCTTGTAC |
| F3 NBS-GFP | ACAAGCACCGGTCCACGTGATGTCGTGCCTAAACGTAATTAACCG |
| R3 NBS-GFP | TAGATATCTCGAGTGCGGCCGCACTTCCTCCTCCTCGGATGC |
| NciI F | AAAACTTTTACAAAAGAATCTTTAATTC |
| NciI R | TAAGTTTGATATTTCGATGCATTC |
| Rec F | AACCAATTCAGTCGACTATGAATCCAACGCTTTCCCG |
| Rec R | ATCTCGAGTGCGGCCGGTCCGGAACCACTTCGTAC |

**A list of gRNA sequences used in this study**

| **Target gene** | **gRNA sequence** |
| --- | --- |
| *rec* | ATCGCGATGGGGCGCCCCCC |
|  | GAATCCAACGCTTTCCCGGG |
|  | AAACATGTACGAAGTGGTTC |
|  | AACGATATAGTGCCAATATT |
| *hdm* | CGACATGCAAGCCATTCCCC |
|  | GGGTGGTCCCATCAAAATAC |
| *PolG1* | TAAGTACTAAGTTGTTACTC |
|  | CGTAGCGGAGGCAATCGAGA |
| *XLF1* | TTCAGTTGAGTAAATCAGTA |
|  | AACAAGTCCTTAGTGATAAA |
| *spn-B* | AAGAAACTCGGCGAAATCAT |
|  | GAATTTAAAATGTGTAACGG |
| *spn-D* | GCGAACGAGTAGATTAATTA |
|  | TTAATTAAGGAATCCGAATG |
| *mre11* | TACTGCCAGCTATGTTATAT |
|  | TTCGTGGTAAACAAGGTCTC |
| *nbs* | GCGATTGAACATCGTCTGTG |
|  | TGCGGCCAGCCACTCGTGCT |
| MCM8 | GATCGATTCATACCATATAA |
|  | CCTGATCTGAGTAAAACCAC |

**A list of primers used for cloning of *Drosophila* DNA repair genes**

| **Gene** | **Forward primer(s)** | **Reverse primer(s)** |
| --- | --- | --- |
| *rec* | AACCAATTCAGTCGACTATGAATCCAACGCTTTCCCG | ATCTCGAGTGCGGCCGGTCCGGAACCACTTCGTAC |
| *Hdm* | GAGCTCGGTACCCGGGGATCATGGCAAGGCGTATTAAGTTCCAGC | ATCTCGAGTGCGGCCGGAAAACGACCATATTTGCGACAAGC |
| *XLF1* | AACCAATTCAGTCGACTATGCTATTGAAAGTAGTGAATATAAATGGCTTGC | ATCTCGAGTGCGGCCGTTTTTGCGCTAACTTATTGATATAGCTC |
| *XRCC4* | AACCAATTCAGTCGACTATGTCCACCTTTGTGGTTAAATTACTTCAACG | ATCTCGAGTGCGGCCGATCTGAGTCGTTATCTATATTCATGACTTGAG |
| *DNA2* | AACCAATTCAGTCGACTGATGGCCACCAAACGCGTGCTG | ATCTCGAGTGCGGCCGGGTGTCCATTAGACCAACCAAATCC |
|  | AACCAATTCAGTCGACTGATGGCCACCAAACGCGTGCTG |  |
| *Pif1* | AACCAATTCAGTCGACTATGGATCTCAATGACGCAG | ATCTCGAGTGCGGCCGGCTAATCGAGACCAGCGGC |
| *mre11* | AACCAATTCAGTCGACTATGAATGGCACCACGACAGCAG | ATCTCGAGTGCGGCCGATCGGAATCATCCGATATAACATAGCTGG |
| *Rrp1* | AACCAATTCAGTCGACTATGCCGCGTGTCAAGGCCG | ATCTCGAGTGCGGCCGTATATTGAAGAATATTGTGATCGGGCA |
| *Spn-A* | AACCAATTCAGTCGACTATGGAGAAGCTAACGAATGTTCAGGC | ATCTCGAGTGCGGCCGGCTCTCCCTGGCGTCTCCTATTCC |
|  | AACCAATTCAGTCGACTATGGCCATTCCCGGCTTGG |  |
| *spn-D* | AACCAATTCAGTCGACTATGAATCATAGATTTGTTCGG | ATCTCGAGTGCGGCCGTCATTCGGATTCCTTAATTAATC |
| *spn-B* | AACCAATTCAGTCGACTATGACAAACTTTCTGGATCAATTGCC | ATCTCGAGTGCGGCCGCAGTCGACGCATTTTGGCGG |
| *parp* | AACCAATTCAGTCGACTATGGATATTGAATTACCTTATCTTGC | ATCTCGAGTGCGGCCGATAAGAATACTTGAATTCCATACG |
| *mms19* | AACCAATTCAGTCGACATGACAACGCCCACGCG | ATCTCGAGTGCGGCCGATTCGGACTAGGCGCACCGACC |
| *CG9601* | AACCAATTCAGTCGACTATGTCTATGGCTAAATACCTAAACC | ATCTCGAGTGCGGCCGTTTCTCGACCAAGTACATTTTGTATAGC |
| *Fen1* | AACCAATTCAGTCGACTATGGGAATTTTGGGCTTATC | ATCTCGAGTGCGGCCGCTTGGGTCGCCGGCCTCGTC |
| *Ercc1* | AACCAATTCAGTCGACTATGGAAGACTTCGACGATGATTCCTTTAACG | ATCTCGAGTGCGGCCGTTTGCTGAGGAACGGTTCCTGC |
| *XLF2* | AACCAATTCAGTCGACATGCTATTGAAAGTAGTGAATATA | ATCTCGAGTGCGGCCGTTTTTTCTCTAGATTATTGATCGCTTC |
| *Okra* | AACCAATTCAGTCGACTCGGCGCAGTCTGGCTCCCAGC | ATCTCGAGTGCGGCCGCTAGAATCCACGGAAGTCC |
| *Rad51D* | AACCAATTCAGTCGACTATGGACCTCAGGCCTATGATTTTGC | ATCTCGAGTGCGGCCGGACCACGCCTGCATCCG |
| *Gen* | AACCAATTCAGTCGACATGGGCGTCAAGGAATTATG | ATCTCGAGTGCGGCCGATCACTAATCACTACCAGGTC |
| *mus81* | AACCAATTCAGTCGACTATGGAGACCCGACTGGAAGTGC | ATCTCGAGTGCGGCCGACGAAAGTGCGTGCTGTAGAATTCG |
| *mei-217* | AACCAATTCAGTCGACTATGTTCACAGTGGATGCAAGCG | ATCTCGAGTGCGGCCGGTTTATTCGTAGATGACATGCTTCCAGTTG |
| *mei-218* | AACCAATTCAGTCGACTATGTCATCTACGAATAAACTAGAGAAAAAGG | ATCTCGAGTGCGGCCGGGGCATGTCCCAGCTATCTGTGC |
| *Rad23* | AACCAATTCAGTCGACTATGTTGACACGCGATTCGTCTAGTTCAAATC | ATCTCGAGTGCGGCCGATCATCGAAGCTAGACGATAGCAAAAAATTAGC |
|  | ATGATTATTACAATTAAAAATCTTCAACAGC |  |
| *CG10694* | AACCAATTCAGTCGACATGAAGCTGTCTATACGCATGC | ATCTCGAGTGCGGCCGGTCTCGATCCTCTTCTGAC |
| *mus312* | AACCAATTCAGTCGACTATGGATCGCAAGACGCGGCGAGCC | ATCTCGAGTGCGGCCGCTTCCTTTTAGAAGGCTTTTTTGTGTGCC |
|  |  | ATCTCGAGTGCGGCCGAAAGAAATCTTCTCCTGCGTAGCGTTGG |
| *Nse1* | AACCAATTCAGTCGACTATGGAGTTGGTGAAACGCG | ATCTCGAGTGCGGCCGACCAATGGAGCGTCTTATTGG |
| *WRNexo* | AACCAATTCAGTCGACATGGAAAAATATTTAACAAAAATGCCC | ATCTCGAGTGCGGCCGCAGAGTCACCTCGTTGATCTTGGTC |
| *mei-9* | AACCAATTCAGTCGACATGGCCGATTCGTGCGCGG | ATCTCGAGTGCGGCCGTCGGCGACCCCTTCGCGATGC |
| *CG14443* | AACCAATTCAGTCGACATGTGTGAGAAAGCTAAGAAACTGG | ATCTCGAGTGCGGCCGATGCATGCGATTGTAGCGTTGC |
| *RecQ5* | ATCTCGAGTGCGGCCGAAACTCACCCTCATACAGATC | ATCTCGAGTGCGGCCGCTTGGATTTTATGAAATATTTTTCAATG |
|  | AACCAATTCAGTCGACATGGCGCATGAAAGCGCTGTGC | ATCTCGAGTGCGGCCGCTCCTCATACAGATCTGCGCAATCC |
| *CG6171* | AACCAATTCAGTCGACATGAGTGCCACAGATGCATCTAC | ATCTCGAGTGCGGCCGCTTACAATCCGCAGGATGGGAG |
| *FancI* | AACCAATTCAGTCGACATGCGGAGTCTGGGTGAGAAG | ATCTCGAGTGCGGCCGTTTCCTTAATGTACGAGGTATTCCC |
| *FancL* | AACCAATTCAGTCGACATGGAAAGTAACGAAGATGTAG | ATCTCGAGTGCGGCCGATCATTTAAAAGTGCCGCAAATG |
| *Xpac* | AACCAATTCAGTCGACATGTCTGCGGAGGTCTCTACC | ATCTCGAGTGCGGCCGCATCTTCTCGTAGGTCTCGCTGTAC |
| *Sws1* | AACCAATTCAGTCGACATGGCGTCCGAGTCAGAAAACAC | ATCTCGAGTGCGGCCGGTCCCGAAAGACGTCCGTCC |
| *tdrd3* | AACCAATTCAGTCGACTATGGAATTAGGCAAGAAACTACG | ATCTCGAGTGCGGCCGATGCTCTCGTTTGTGGGGCG |
| *Pcm* | AACCAATTCAGTCGACTATGGGCGTTCCCAAGTTCTTTCG | ATCTCGAGTGCGGCCGAGTTGGATGCGGGGAGTCG |
| *CG30094* | AACCAATTCAGTCGACATGGCAGACGATCCAAACAAGC | ATCTCGAGTGCGGCCGTTTTCCGTTCTTCTTTTTGG |

**A list of primers used for RT-qPCR and PCR**

| **Gene** | **Forward Primer** | **Reverse primer** |
| --- | --- | --- |
| *rec* | TTGGCGCAGGGCAACTTCTT | GACTGACCACGTGGGATAAAT |
| *ef1a* | GCGTGGGTTTGTGATCAGTT | GATCTTCTCCTTGCCCATCC |
| *TFAM* | GGAGCTCTACAGGAAAGCGATTTCC | CGATAAGATTTCCGTGACGCACC |
| *twinkle* | AAGCGGACGGGAGCCTTTATC | CAACCGGCTTGGGCAACTGATAG |
| *mtSSB* | TTTTCGGTTGCTACACACA | TGAACACCACTACACGATG |
| *PolG1* | TGGAGGACAGGGCCTACAG | TCTTCCAGGCGATTGAACATG |
| *cox4* | GAACATCTTCGTGTACGATGAGC | GGTTGATTTCCAGGTCGATGATGC |
| *mt:CoI* | GCTATTGGATTATTAGGATTTATTGT | TCCTAAAGCTCATAAAATAGCTGGAGA |
| *Human mtDNA* | CGGCGCATGAGCTGGAGTCCTAG | CTAGTCAGTTGCCAAAGCCTCCG |
| *Human nuclear DNA* | AGAAGAGCTACGAGCTGCCTGAC | GGAGTTGAAGGTAGTTTCGTGGATG |

**A list of primers used to amplify *Drosophila* mtDNA**

| **Primer name** | **Sequence** |
| --- | --- |
| F1741 | ATAGCATTCCCACGAATAAATAATATAAG |
| F3251 | CATGGACAACTTATTGAAATAATTTG |
| F5561 | GTATATTTGACTTCCAATCATAAGGTC |
| R4564 | CCAGCAATTATATTAGCAGTTAATCG |
| R7509 | GCTGCTCCTACACCTGTTTCTGC |
