# Supplementary figures and images for: REC drives recombination to repair double-strand breaks in animal mtDNA
Source: J Cell Biol. 2022 Nov 10;222(1):e202201137. doi: 10.1083/jcb.202201137 (PMC9652705; doi:10.1083/jcb.202201137)

Figure 1D

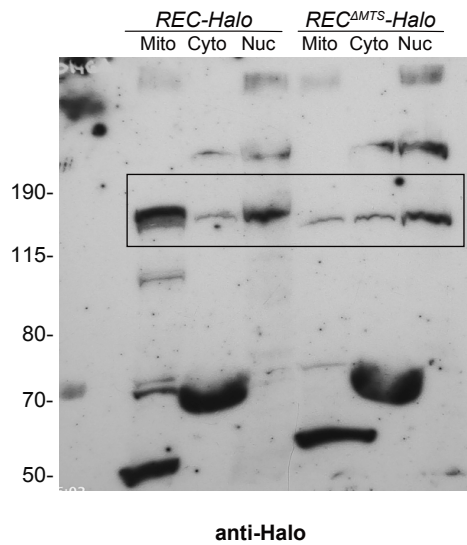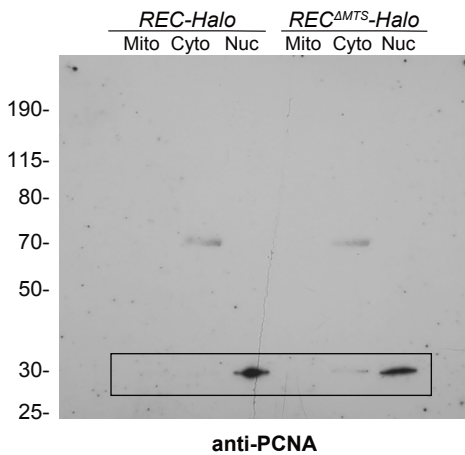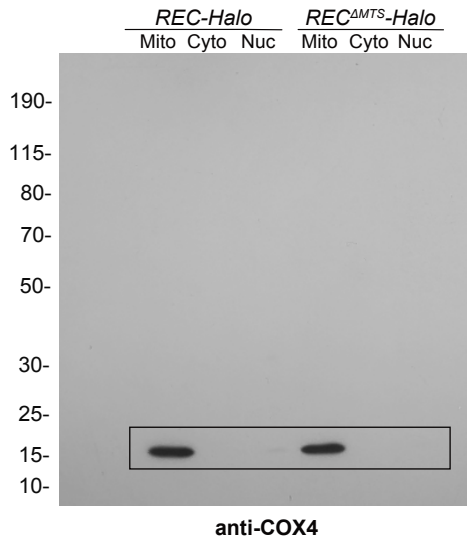

Figure 1E

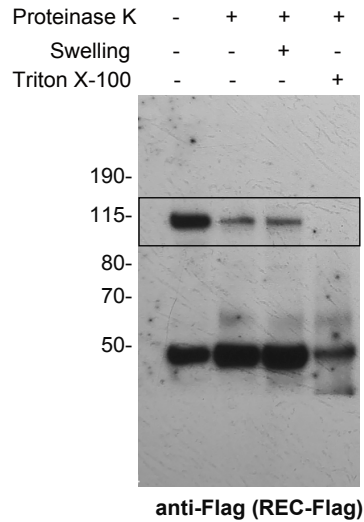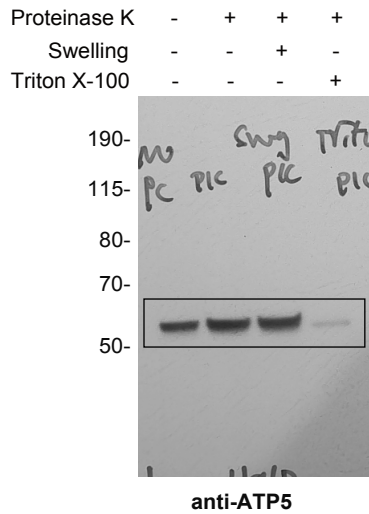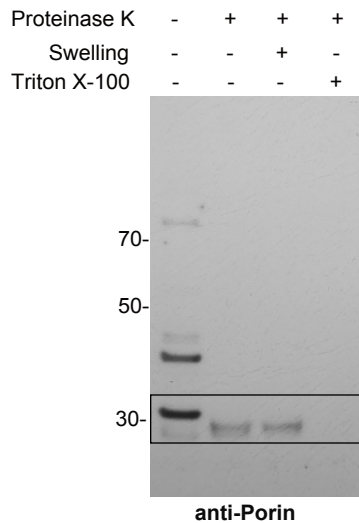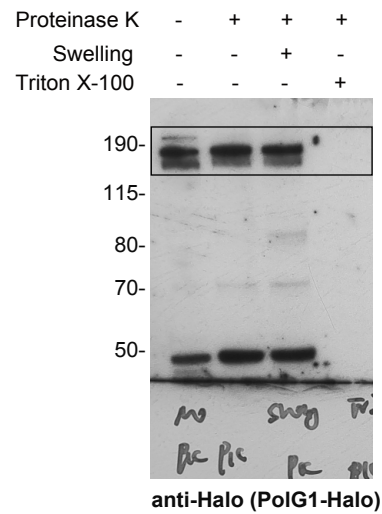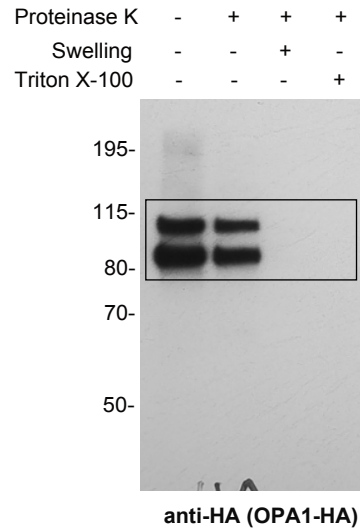

Supplement: SourceData F1 — is the source file for Fig. 1. [file JCB_202201137_SourceDataF1.pdf]

Figure 3B

Repeat 1

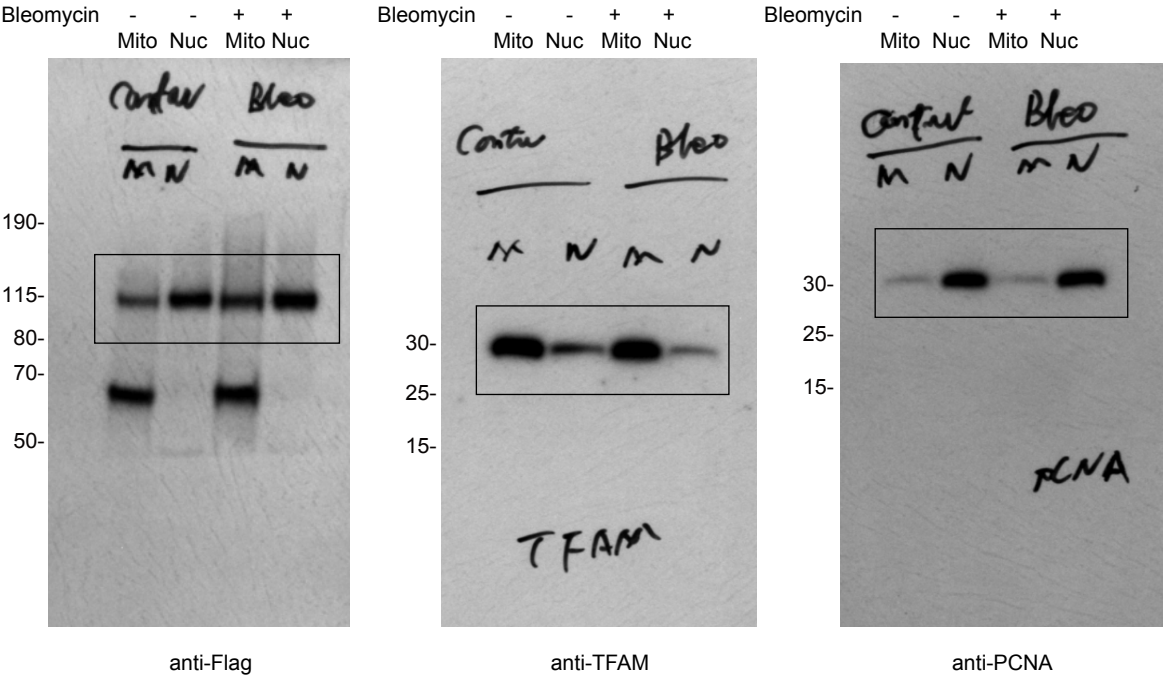

Repeat 2

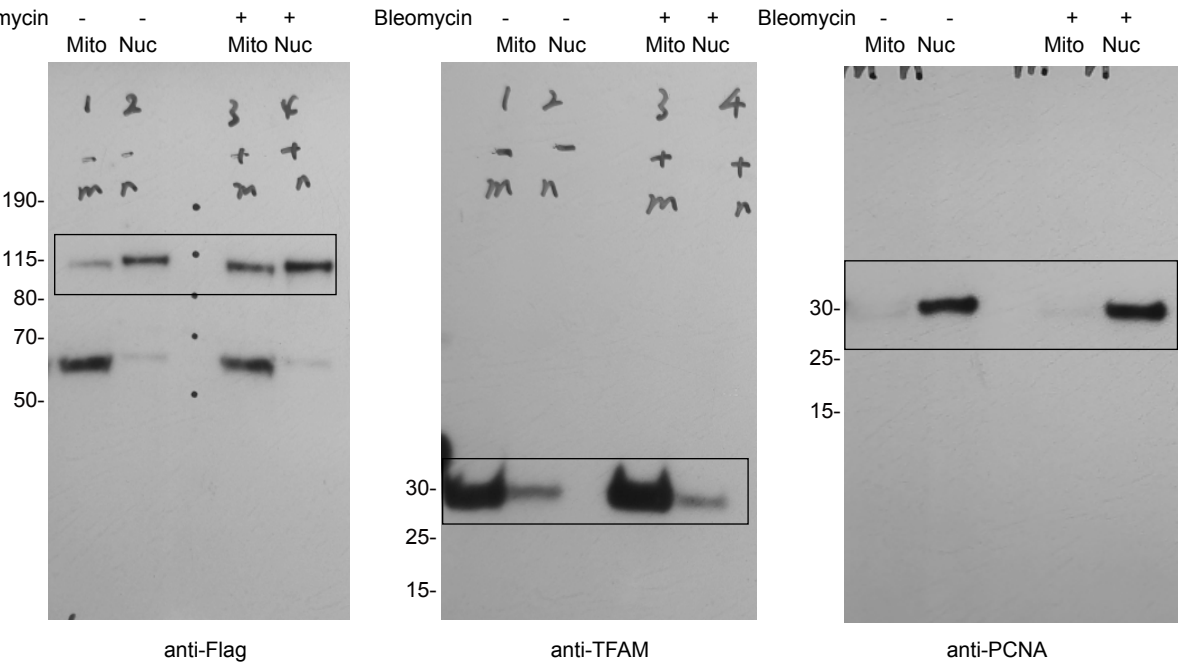

Repeat 3

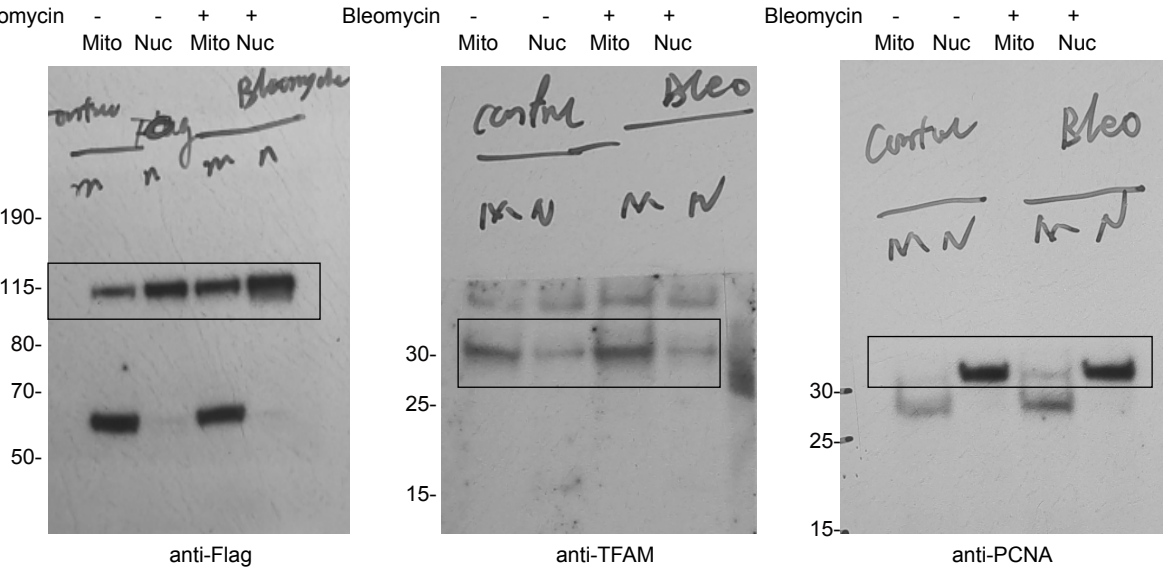

Supplement: SourceData F3 — is the source file for Fig. 3. [file JCB_202201137_SourceDataF3.pdf]

Figure 5B

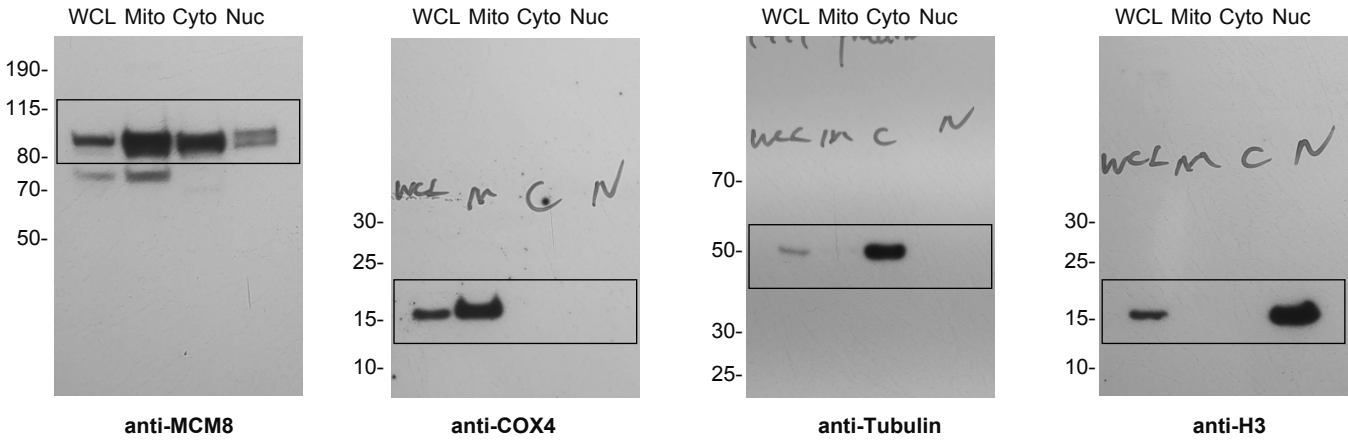

Figure 5C

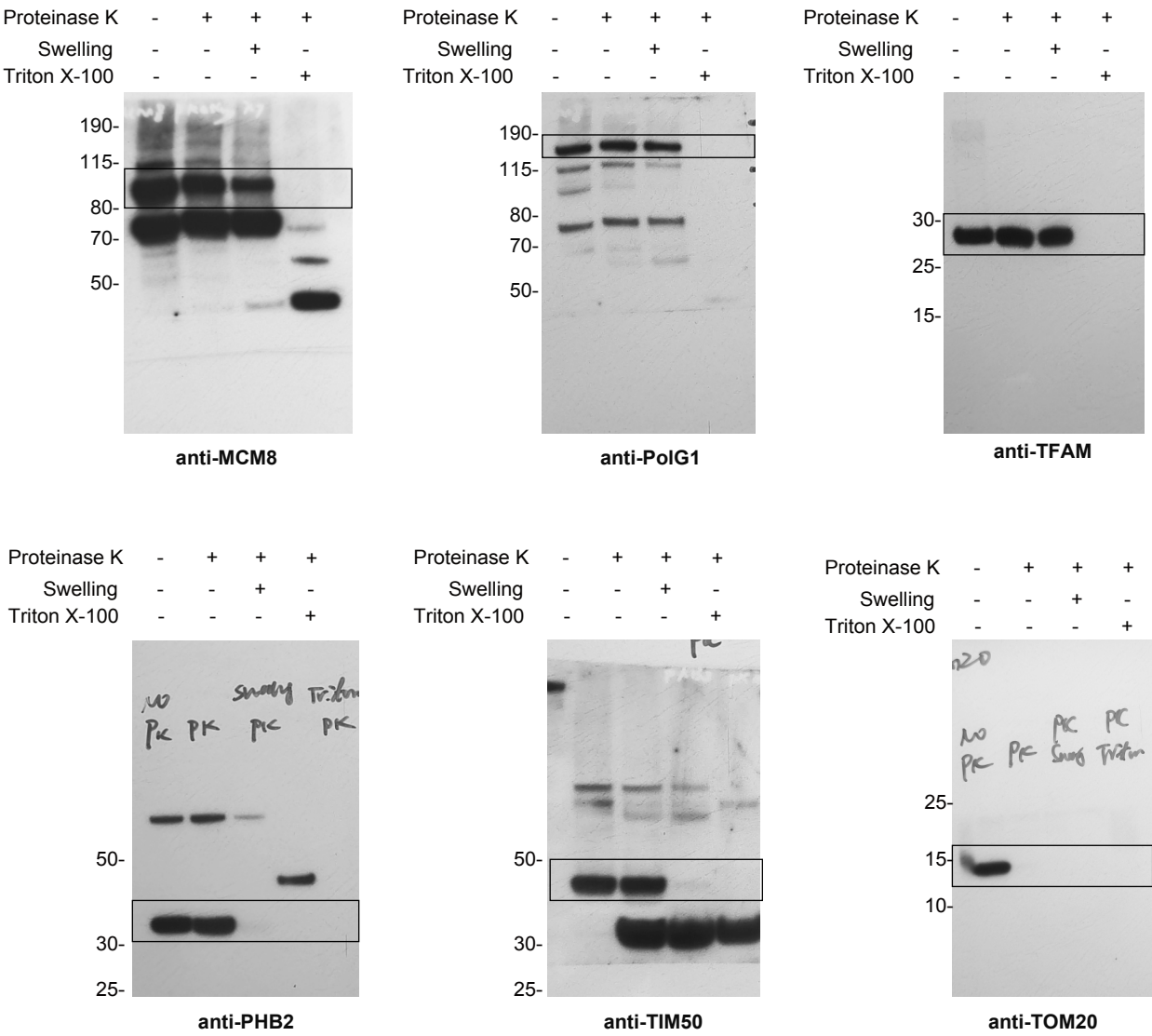

Figure 5D

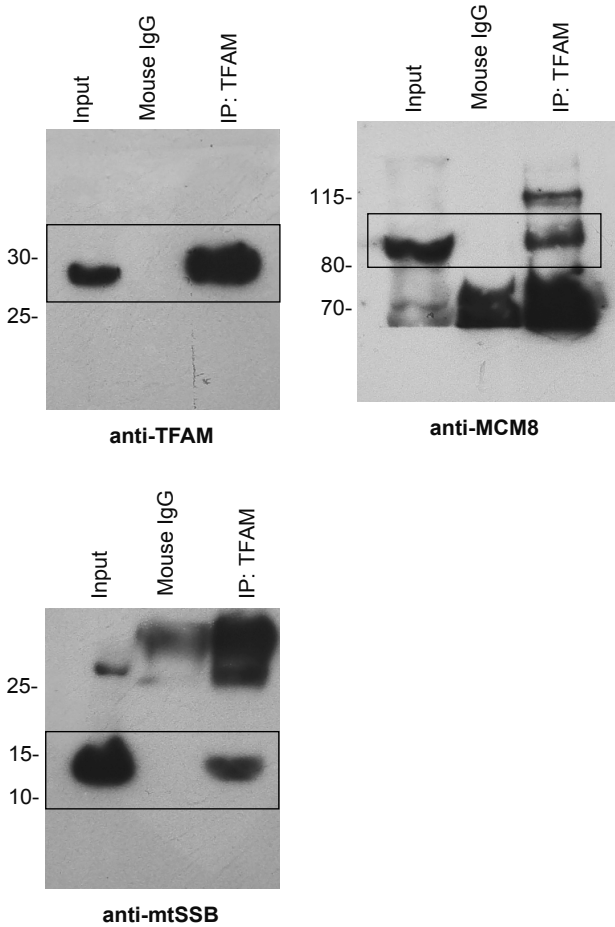

Figure 5E

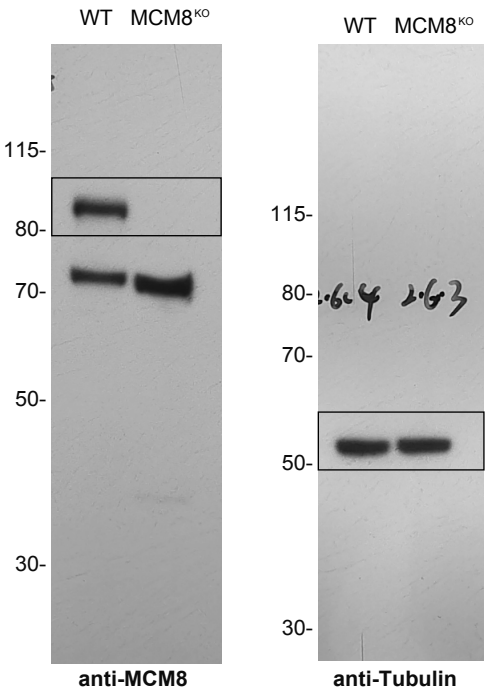

Supplement: SourceData F5 — is the source file for Fig. 5. [file JCB_202201137_SourceDataF5.pdf]

Figure S1E

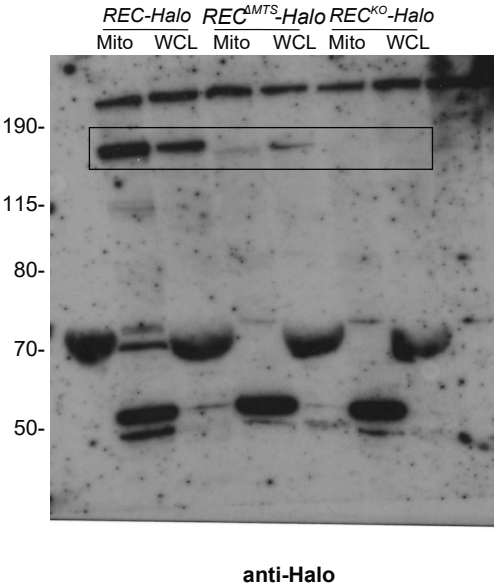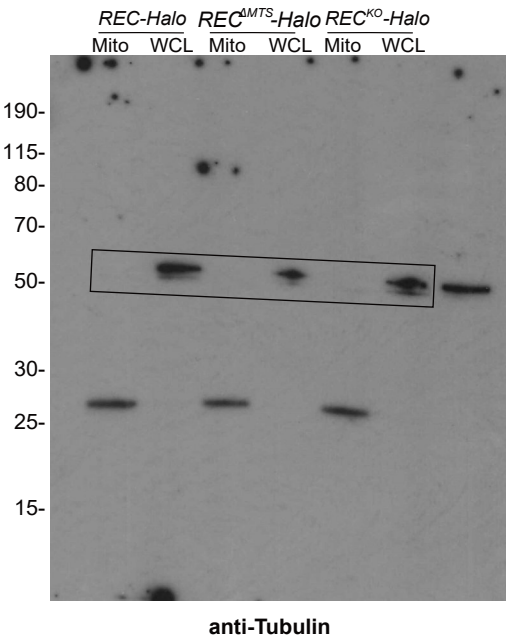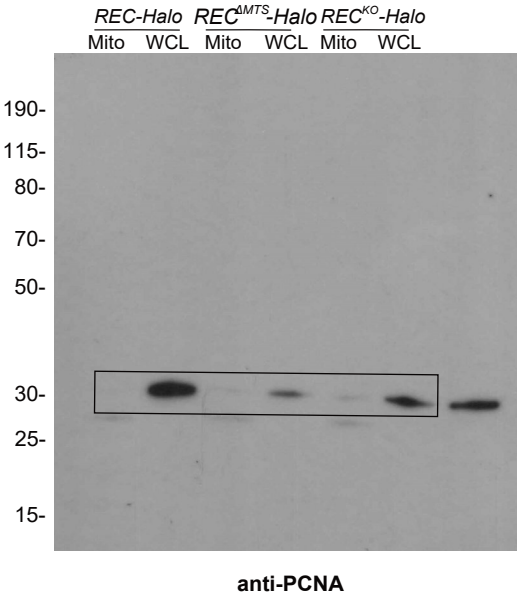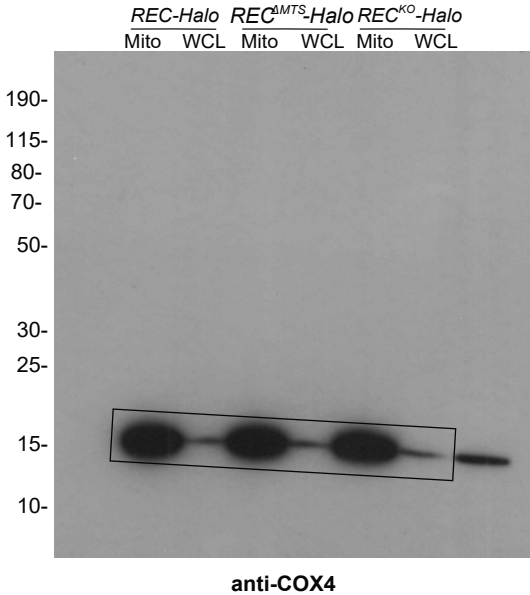

Supplement: SourceData FS1 — is the source file for Fig. S1. [file JCB_202201137_SourceDataFS1.pdf]

Figure S4C

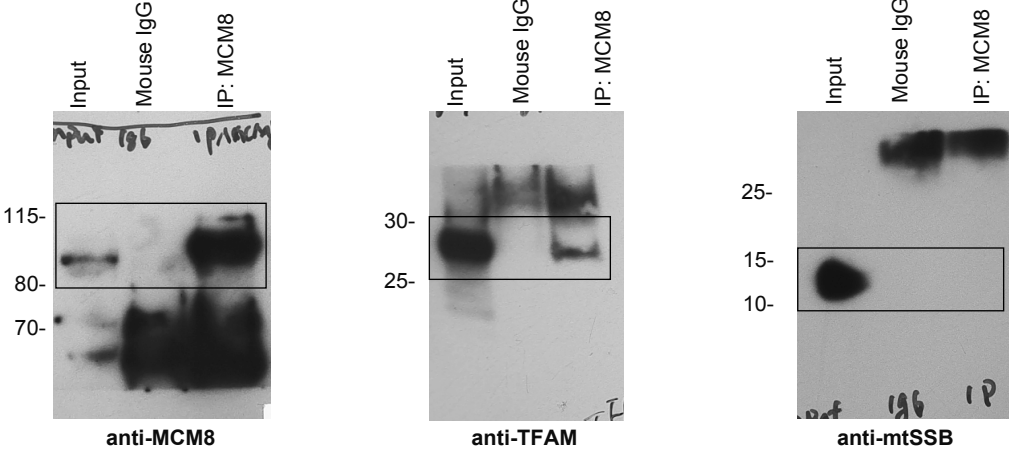

Figure S4D

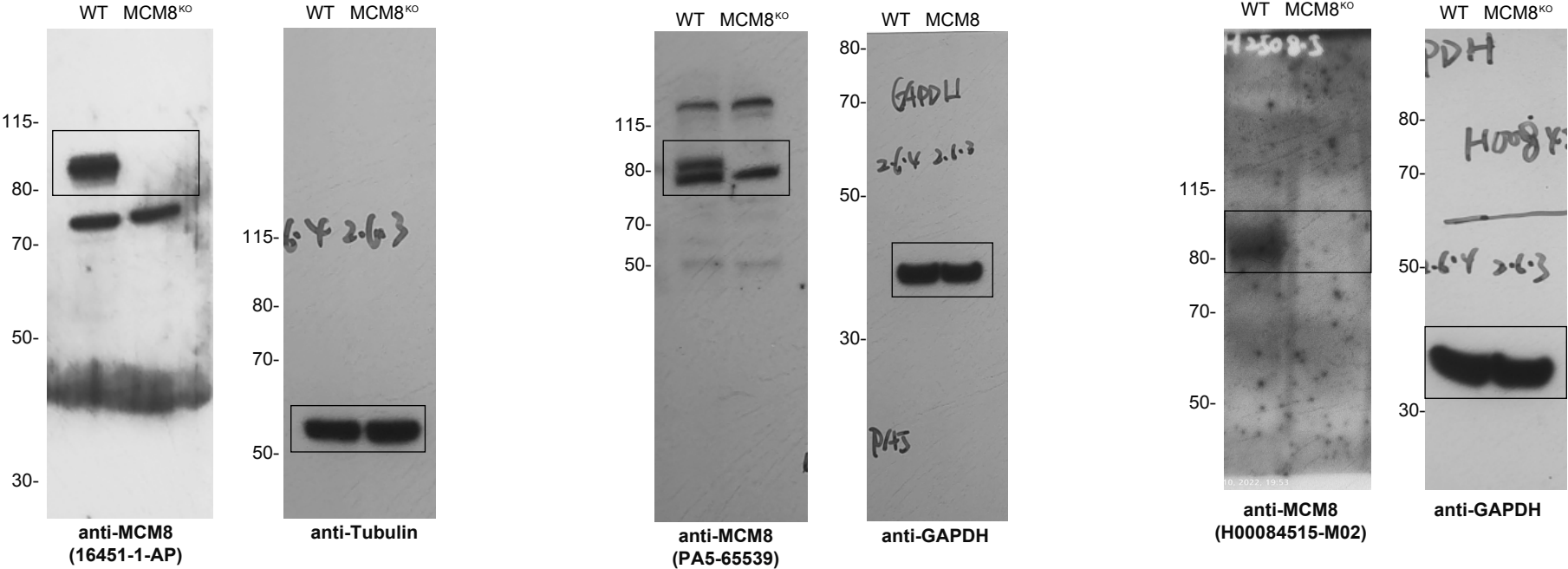

Supplement: SourceData FS4 — is the source file for Fig. S4. [file JCB_202201137_SourceDataFS4.pdf]
